# Association between plant-based diets and the risk of coronary heart disease predicted using the Framingham Risk Score in Korean men: data from the HEXA cohort study

**DOI:** 10.4178/epih.e2024035

**Published:** 2024-02-28

**Authors:** Khongorzul Ganbat, Bayarmaa Nasan Ulzii, Sangah Shin

**Affiliations:** Department of Food and Nutrition, Chung-Ang University, Anseong, Korea

**Keywords:** Diet, Plant-based, Coronary disease, Cohort studies

## Abstract

**OBJECTIVES:**

This study investigated the potential correlation between 4 plant-based diet indices and the predicted risk of coronary heart disease (CHD) in Korean men using the Framingham Risk Score.

**METHODS:**

The study included 12,356 men participants (aged ≥40 years) from the Health Examinees Study. Dietary intake was estimated using a validated food frequency questionnaire. Four plant-based diet indices were measured, including the overall plant-based diet index, the healthy plant-based diet index (hPDI), the unhealthy plant-based diet index (uPDI), and the pro-vegetarian diet index. Multivariable Cox proportional hazard models were used to estimate hazard ratios (HRs) with 95% confidence intervals (CIs) for the predicted 10-year risk of CHD.

**RESULTS:**

The study found that individuals in the highest hPDI quintile had a 19% lower risk score for CHD based on the Framingham Risk Score (model 3: HR, 0.80; 95% CI, 0.69 to 0.93; p for trend=0.010). In stratified analyses, the highest pro-vegetarian diet index was associated with a lower risk score for CHD in physically active individuals (HR, 0.74; 95% CI, 0.59 to 0.93; p for interaction=0.020). Conversely, the highest uPDI was associated with the highest risk score for CHD in those with a body mass index of ≥25 kg/m^2^ and a waist circumference ≥90 cm.

**CONCLUSIONS:**

This prospective cohort study highlights the positive role of adhering to a high hPDI diet in the prevention of CHD in Korean men. Further prospective studies are needed to determine the association between various plant-based diet indices and the risk of CHD in Asian populations with different dietary habits.

## GRAPHICAL ABSTRACT

[Fig f4-epih-46-e2024035]

## Key Message

The study highlights the important role of dietary patterns, especially healthy plant-based diet index (hPDI), in reducing the risk of coronary heart disease (CHD) among Korean men. A significant 19% reduction in CHD risk associated with the highest hPDI underscores the potential impact of dietary choices on cardiovascular health.

## INTRODUCTION

Coronary heart disease (CHD), a leading cause of mortality worldwide, is more prevalent in men than in women [1]. According to the World Health Organization, cardiovascular diseases (CVDs) accounted for approximately 32% (17.9 million people) of global deaths in 2019 [2]. In Korea, CVDs are the second leading cause of death based on the Cause of Death Statistics in 2021 [3]. Korean adults exhibit multiple cardiovascular risk factors, including obesity, diabetes, hypercholesterolemia, hypertension, and smoking, which tend to rise with increasing age [4]. In Korean adults aged ≥ 30 years, hypertriglyceridemia is twice as prevalent in men than in women. Moreover, exposure to various chronic disease risk factors increases with age [5]. As the population of older individuals with multiple risk factors and chronic diseases has grown, CHD prevention has become a crucial issue in Korea. Gender differences in the prevalence of myocardial infarction are attributable to several factors, including biological, lifestyle, and social factors [6]. Higher testosterone levels in men can contribute to increased plaque buildup, whereas women benefit from the protective effects of estrogen. When compared with women, men often have higher levels of harmful visceral fat, different metabolic profiles, and an increased susceptibility to smoking-related risks [7]. In addition, men tend to have higher blood pressure and cholesterol levels, further elevating the likelihood of CHD [8].

The common risk factors for CHD include hypertension, diabetes, obesity, dyslipidemia, and family history, and many CHD risk factors are influenced by lifestyle choices [9]. A healthy diet and lifestyle are considered the most effective methods for preventing CHD. Previous studies have shown that diets rich in plant-based foods reduce lipid levels, suggesting that plant-based diets are associated with a lower CHD risk [10-12]. Plant-based diets typically involve a lower intake of animal foods and a higher intake of plant foods [13,14]. However, not all plant-based foods are healthy, and some are associated with a higher risk of metabolic disorders. For instance, refined grains, potatoes, and sugar-sweetened beverages have been associated with an increased risk of chronic diseases, such as type 2 diabetes, obesity, and CVD [15-17].

Recently, plant-based diet indices have been developed to assess plant and animal food consumption, as well as the quality of plant foods. The overall plant-based diet index (PDI) reflects the ratio of plant foods to animal foods in the diet, with higher scores indicating a greater intake of plant foods relative to animal foods [18- 20]. The healthy plant-based diet index (hPDI) evaluates diets that emphasize a higher consumption of healthy plant foods and a lower intake of less healthy plant and animal products [11,18,19]. The pro-vegetarian diet index promotes a higher consumption of selected plant foods and a lower intake of animal foods [21]. The unhealthy plant-based diet index (uPDI) evaluates diets with a higher intake of less healthy plant foods and lower consumption of healthy plant and animal foods [18,20,22]. However, cohort study-based evidence regarding the association between plantbased diets and CHD in the Asian population in general and among Korean adults in particular is limited. Therefore, this study assessed the association between the 4 plant-based diet indices (PDI, hPDI, uPDI, and pro-vegetarian diet index) and the predicted risk of CHD in Korean adults using data from the Health Examinees (HEXA) study.

## MATERIALS AND METHODS

### Study population

**Figure 1.**
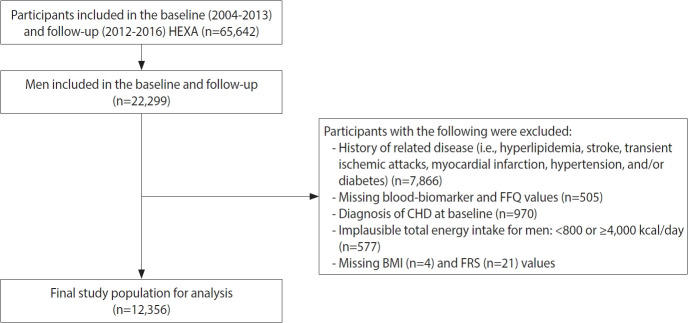
Flow chart of participants. HEXA, Health Examiness; FFQ, food frequency questionary; CHD, coronary heart disease; BMI, body mass index; FRS, Framingham Risk Score.

The HEXA study is a prospective large-scale genomic cohort survey of the Korean population. The baseline HEXA study included 173,357 participants who underwent health check-ups and completed a series of questionnaires between 2004 and 2013. Among them, 65,642 participants were followed up from 2012 to 2016. The study involved participants (aged 40-79 years) from 38 general hospitals and health examination centers located in 8 regions of Korea [23,24]. A total of 22,299 men participants who completed baseline and follow-up assessments were included in these analyses. The median follow-up duration was 4.2 years. However, we excluded participants with hyperlipidemia, stroke, transient ischemic attacks, myocardial infarction, hypertension, and diabetes at baseline (n= 7,866) or those with missing blood biomarker values and food frequency questionnaire (FFQ) data (n= 505). Furthermore, we excluded those with a CHD diagnosis at baseline (n=970), implausible energy intake ( < 800 or ≥ 4,000 kcal/day, n = 577) [25], or those lacking body mass index (BMI) data (n= 4) and Framingham Risk Score (FRS) parameters (n= 21). In this study, the final analyses included 12,356 participants (Figure 1).

### Dietary assessment and calculation of the 4 plant-based diet indices

Using a semi-quantitative FFQ, 106 food items were used to collect information about dietary intake. Previous research [26] has described the reproducibility and validity of the FFQ in detail. Participants were asked how much (i.e., portion size) and how frequently (on average) they had consumed each food item in the previous year. The questionnaire categorized each food item into 9 frequency options: never or seldom, once a month, 2-3 times a month, once or twice a week, 3-4 times a week, 5-6 times a week, once a day, twice a day, and 3 times a day. The portion of each food item was classified as small, medium, or large. The nutrient intake of each food item was calculated using Korean standard food composition tables [27]. The long-term intake of the 106 food items was assessed using baseline and follow-up FFQs to estimate the cumulative average consumption [27].

We measured the following plant-based diet indices: PDI, hPDI, uPDI, and the pro-vegetarian diet index. Previous studies have described the calculation of these diet indices in detail [11,20,28]. Food items were categorized into 17 food groups in the PDI, hPDI, and uPDI and 11 food groups in the pro-vegetarian diet index. The 17 food groups were then classified into 3 main groups: healthy plant foods (such as whole grains, fruits, vegetables, nuts, legumes, tea, and coffee), less-healthy plant foods (such as refined grains, potatoes, sugar-sweetened beverages, sweets and desserts, and salty plant foods), and animal foods (such as animal fat, dairy, eggs, fish or seafood, meat, and miscellaneous animal foods). Salty foods, such as kimchi and pickled vegetables, were classified as less healthy plant foods. The 11 food groups were classified into plant foods (e.g., grains, fruits, vegetables, nuts, legumes, and potatoes) and animal foods (e.g., animal fat, dairy, eggs, fish or seafood, and meat) (Supplementary Material 1). However, we did not separate fruits and fruit juices because they were surveyed together in the FFQ.

The participants were ranked into quintiles based on the energy-adjusted consumption of each food group (17 or 11) [18,29]. Positive or negative scores were assigned to all food groups. For the PDI, all plant food groups were given positive scores, while animal food groups were given negative scores. For example, participants in the highest quintile of “nuts” (a plant food group) received a score of 5, while those in the lowest quintile received 1 point. Conversely, participants in the highest quintile of “fish or seafood” (an animal food group) had a score of 1, while those in the lowest quintile had a score of 5. For the hPDI, only the healthy food groups received positive scores, whereas the less-healthy plant and animal food groups were given negative scores. For the uPDI, the less-healthy plant food groups were assigned positive scores, while the healthy plant food groups and animal food groups were assigned negative scores. The pro-vegetarian diet index was scored like the PDI except that some food groups, such as tea and coffee, salty plant foods, sugar-sweetened beverages, sweets and desserts, and miscellaneous animal foods, were not scored [25]. Finally, we summed up the scores of all individual food groups and divided the participants into quintiles, based on their 4 index scores, for analysis. The PDI, hPDI, and uPDI scores ranged from 17-85, and the pro-vegetarian diet index scores ranged from 11-50. All participants were given written informed consent forms before taking part in the study.

### Definition of coronary heart disease risk

CHD risk was defined based on the FRS. Participants deemed to be high risk at the start (based on baseline FRS) were excluded from the study, and the remaining participants were categorized into CHD risk groups based on their follow-up FRS scores. The FRS algorithm was developed to predict the 10-year risk of CHD using data on factors like age, smoking status, diabetes status, blood pressure, total cholesterol (TC), and high-density lipoprotein cholesterol (HDL-C) at baseline and during follow-up [30]. A previous study compared FRSs based on low-density lipoprotein cholesterol (LDL-C) versus TC and found that TC was a more appropriate indicator in Korean adults [31]. The outcome of this study was a 10-year CHD risk of ≥ 20%, which was used as an indicator of the predicted risk of CHD only in the analyses of follow-up data.

### Assessment of covariates

The baseline data, including socio-demographic characteristics like age, gender, education level, and lifestyle factors, were collected using a self-reported questionnaire. BMI was calculated by dividing weight (kg) by height squared (m^2^). Weight was categorized as underweight (BMI < 18.5 kg/m^2^), normal weight (BMI ≥ 18.5 to < 23.0 kg/m^2^), overweight (BMI ≥ 23.0 to < 25.0 kg/m^2^), and obese (BMI ≥ 25.0 kg/m^2^). Education level was categorized as middle school, high school, and college. Household income was categorized into groups earning <3 million Korean won and ≥3 million Korean won per month. Regarding drinking status, participants were classified as current drinkers or non-drinkers (never consumed alcohol or consumed alcohol in the past). Regarding smoking status, participants were classified as non-smokers, past smokers, or current smokers. Finally, for physical activity they were classified as active (exercise ≥ 30 min/day) or inactive, based on responses to the questions: (1) Do you regularly exercise to the point you are sweating? and (2) Is your exercise frequency at least 5 days per week? [32].

### Statistical analysis

We examined the differences in baseline characteristics and lifestyle factors according to the quintiles of the 4 indices using the chi-square test for categorical variables and a generalized linear regression model for continuous variables. Categorical variables are presented as numbers and percentages, whereas continuous variables are presented as means and standard deviations.

Follow-up person-years were computed for each participant, spanning the period from baseline to follow-up examination. Multivariable Cox proportional hazards regression analysis was used to estimate the hazard ratio (HR) and 95% confidence interval (CI) for each index and the predicted risk of CHD. The Cox proportional hazards assumption was assessed using Schoenfeld residuals [33]. Model 1 was adjusted for age (year, continuous) and BMI (continuous). In model 2, additional adjustments were made for education level, smoking status, drinking status, household income level, physical activity, and total energy intake (continuous) [34]. Model 3 was adjusted for age (continuous), waist circumference (WC; continuous), education level, smoking status, drinking status, household income level, physical activity, and total energy intake (continuous). To assess potential effect modifiers, we conducted stratified analyses based on participant characteristics, including age (median age < 52 and ≥ 52 years), BMI (< 25.0 and ≥ 25.0 kg/m^2^) [34], WC (< 90 and ≥ 90 cm), smoking status (nonsmoker, past smoker, and current smoker), drinking status (nondrinker and current drinker), and physical activity (active and inactive). The association between the plant-based diet indices and the predicted risk of CHD was examined using restricted cubic splines with 4 knots. Trend tests were performed with a generalized linear model using the median value of each group. The median value was modeled as a continuous variable. The interaction p-values were calculated using a likelihood ratio test to compare the Cox proportional hazard models with and without cross-product terms for CHD risk. All data analyses were performed using SAS version 9.4 (SAS Institute Inc., Cary, NC, USA). Based on 2-sided tests, p-value< 0.05 indicated statistical significance.

### Ethics statement

The HEXA study protocol was approved by the Institutional Review Board of the Ethics Committee of the Korea National Institute of Health for the Korean Genome and Epidemiology Study (IRB No. E-1503-103-657).

## RESULTS

Baseline data were collected from 2004 to 2013, and follow-up surveys were conducted from 2012 to 2016. During the follow-up (average: 4.2 years), 2,017 were predicted to be at risk for CHD. The general characteristics of the participants at baseline are listed in Table 1 according to PDI quintiles. Participants in the highest PDI quintile were older, had higher education levels, and were more likely to be non-drinkers, non-smokers, and physically active. Similarly, participants in the highest quintile of the hPDI and pro-vegetarian diet index were more likely to be non-drinkers, non-smokers, and physically active (Supplementary Materials 2 and 3). However, participants in the highest uPDI quintile were more likely to have lower education levels, be current smokers, and be physically inactive (Supplementary Material 4, all: p< 0.05).

Baseline data for the daily nutrient intake of participants according to the quintiles of each index are shown in Table 2. For all indices, participants in the highest quintiles had higher percentages of energy intake from carbohydrates, lower percentages of energy intake from protein and fat, and a lower intake of cholesterol. Moreover, participants in the highest quintile of the PDI, hPDI, and pro-vegetarian diet index had higher fiber intake, while those in the highest uPDI quintile had lower fiber intake. Participants in the highest hPDI quintile had lower sodium intake when compared with other hPDI quintiles (all: p< 0.05).

The participants’ baseline blood biomarker data according to the quintiles of each index are shown in Table 3. Participants in the highest PDI, hPDI, and pro-vegetarian diet index quintiles had lower TC, triglyceride, and LDL-C levels. In addition, participants in the highest PDI and pro-vegetarian diet index quintiles had lower fasting blood glucose levels. However, individuals in the highest uPDI quintile had higher triglyceride levels and lower TC and HDL-C levels (all: p< 0.05).

**Figure 2.**
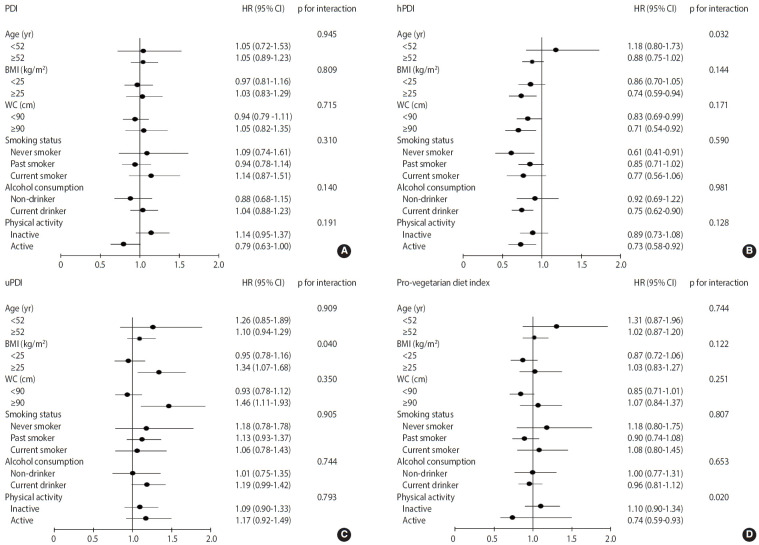
Stratified analysis of the association between plant-based diet indices and CHD. (A) Overall plant-based diet index (PDI). (B) Healthy plant-based diet index (hPDI). (C) Unhealthy plant-based diet index (uPDI). (D) Pro-vegetarian diet index. The models were adjusted for age, BMI, WC, total energy intake, smoking status, education level, household income level, and physical activity. HR, hazard ratio; CI, confidence interval; BMI, body mass index; WC, waist circumference.

**Figure 3.**
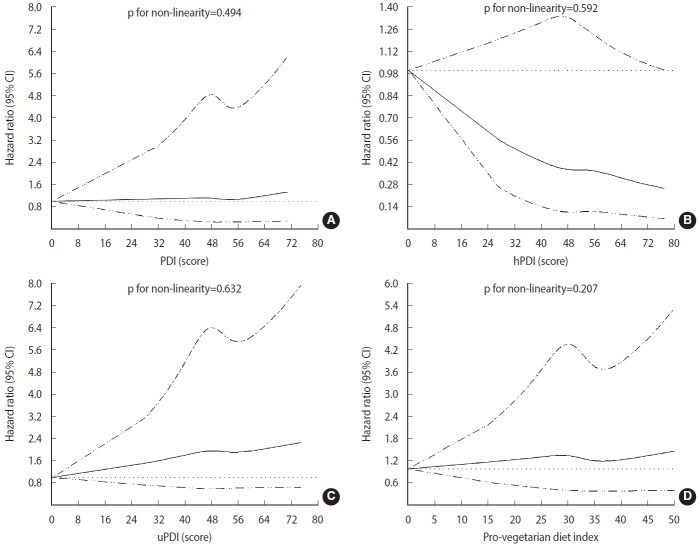
Adjusted restricted cubic spline models for assessing the associations between plant-based diet indices and CHD. (A) Overall plant-based diet index (PDI). (B) Healthy plant-based diet index (hPDI). (C) Unhealthy plant-based diet-index (uPDI). (D) Pro-vegetarian diet index. The models were adjusted for age, body mass index, total energy intake, smoking status, educat ion level, household income level, and physical activity. The 4 knots set: 5th, 35th, 65th, 95th percentiles of each score. The black solid lines represent hazard ratio, and the dashed lines represent the 95% confidence intervals (CI).

The ranges of the 4 indices were 32-71, 27-77, 28-75, and 15-50 for the PDI, hPDI, uPDI, and pro-vegetarian diet index, respectively (Table 4). The HRs and 95% CIs of the predicted risk of CHD based on each index are also presented in Table 4. In model 1 (ageand BMI-adjusted), the PDI (HR, 0.86; 95% CI, 0.75 to 0.98; p for trend= 0.017), hPDI (HR, 0.75; 95% CI, 0.65 to 0.86; p for trend = 0.001), and pro-vegetarian diet index (HR, 0.84; 95% CI, 0.73 to 0.97; p for trend = 0.007) were inversely associated with the predicted risk score for CHD. However, the uPDI did not show a significant association with the risk of CHD. In model 2, where further adjustments were made for other factors, the highest hPDI quintile, when compared with the lowest quintile (HR, 0.81; 95% CI, 0.69 to 0.94; p for trend= 0.013) and model 3 (HR, 0.80; 95% CI, 0.69 to 0.93; p for trend= 0.010), showed a 19% lower CHD risk. However, the PDI, uPDI, and pro-vegetarian diet index were not significantly associated with the predicted risk of CHD. Stratified analyses were conducted based on age, BMI, smoking status, alcohol intake, and physical activity (Figure 2). This revealed that among individuals with high adherence to the hPDI, those with a BMI of ≥ 25.0 kg/m^2^, non-smokers, current alcohol drinkers, and physically active individuals had a lower risk score for CHD when compared with those in the lowest hPDI quintile. Stratified analyses identified meaningful interactions between the pro-vegetarian diet index and uPDI subgroups. Among physically active individuals, a higher pro-vegetarian diet index was linked to a reduced risk score for CHD (HR, 0.74; 95% CI, 0.59 to 0.93; p for interaction= 0.020). Conversely, the highest uPDI level was associated with higher CHD risk scores in those with BMIs ≥ 25.0 kg/m² (HR, 1.34; 95% CI, 1.07 to 1.68; p for interaction= 0.040) and WC values ≥ 90 cm (HR, 1.46; 95% CI, 1.11 to 1.93; p for interaction=0.350). In addition, higher hPDI levels indicated a lower CHD risk in both WC groups. A potentially stronger protection was suggested in those with higher WC, but further research is needed to confirm this difference. For sensitivity analyses (Supplementary Material 5), model 4 was further adjusted for other factors, such as a comparison of animal food intake in the highest quintile of the hPDI with the lowest quintile (HR, 0.80; 95% CI, 0.68 to 0.93; p for trend= 0.010). In model 5, further adjustments were also made for other factors, including energy from carbohydrates, protein, and fat (HR, 0.80; 95% CI, 0.68 to 0.94; p for trend= 0.013). Restricted cubic spline analysis was used to examine the non-linear relationship between the 4 indices and the predicted risk of CHD. The spline curves with 4 knots set at the 5th, 35th, 65th, and 95th percentiles of each score are shown in Figure 3. The models were adjusted for age, BMI, total energy intake, education level, smoking status, drinking status, household income level, and physical activity. However, no significant non-linear correlation was observed between the plant-based diet indices and the predicted risk of CHD (p_non-linearity_ > 0.05).

## DISCUSSION

This prospective cohort study of Korean adult men found an association between higher adherence to the hPDI and a 19% lower risk score for CHD after adjusting for socio-demographic and lifestyle factors. This finding is consistent with previous studies involving Western populations that also associated a higher hPDI with a lower CHD risk.

Previous studies have examined the association between plant-based diet indices and other cardiovascular risk factors. Prospective cohort studies have reported that higher hPDI levels are associated with a lower CHD risk [11]. The Atherosclerosis Risk in Communities study, which involved middle-aged adults in the United States, found that the highest quintile of the PDI, hPDI, and pro-vegetarian diet index was associated with a lower risk of hypertension [20]. The Henan Rural Cohort Study involving Chinese adults reported that a higher PDI was associated with a lower risk of type 2 diabetes [35]. The North West Adelaide Health Cohort Study showed that higher adherence to the hPDI was associated with a lower risk of obesity [36]. However, it is worth noting that some cohort studies involving Korean adults did not find an association between hPDI and the risk of metabolic syndrome [28,37].

The current study indicates that the nutritional composition of the hPDI may decrease the predicted risk of CHD. hPDI-adherent diets contain a higher proportion of whole grains, fruits, vegetables, legumes, tea, and coffee and are associated with a lower intake of refined grains, sugar-sweetened beverages, animal fat, and meat. These diets have a lower intake of total energy and sodium and a higher consumption of fiber, antioxidants, and micronutrients when compared with other plant-based diets. Several mechanisms may explain the association between healthy plant-based diets and CHD. A meta-analysis associated higher dietary fiber intake with a lower incidence of early mortality and CVDs [38]. For instance, a higher fiber intake decreases the serum concentration of inflammatory markers, such as C-reactive protein [39]. Fiber positively impacts blood lipid profiles by reducing the serum levels of TC, triglycerides, and oxidized low-density lipoproteins [40]. Previous studies have shown that polyphenols improve the lipid profile, limit oxidized low-density lipoprotein, and reduce vascular inflammation [41,42]. In addition, a healthy plant-based diet is rich in antioxidant nutrients like vitamin C, vitamin E, and beta-carotene. Potassium helps reduce blood pressure and the incidence of stroke [43], while magnesium improves cardiometabolic outcomes by affecting glucose metabolism and insulin sensitivity [44,45]. Furthermore, a healthy plant-based diet improves the composition of gut microbiota. For instance, fibers and polyphenols increase the abundance of some probiotics, such as *Bifidobacterium* and *Lactobacillus*, which have anti-pathogenic and anti-inflammatory effects [46].

We did not find associations between CHD risk and the PDI, pro-vegetarian diet index, and uPDI. This may be attributed to the high consumption of plant-based foods by the Korean population, regardless of the overall quality of the foods. In traditional Korean diets, plant foods are consumed in higher amounts, and vegetables are commonly included in all meals as side dishes [47,48]. Therefore, increasing the consumption of nutritious plant foods may not provide additional benefits that prevent CHD in a population already accustomed to plant-based diets. In addition, the lack of association with uPDI may be due to the categorization of foods in our study, which did not always distinguish between less-healthy plant foods and healthy plant foods. For instance, fruit juices were included in the “fruits” category.

Studies in Japan and China have indicated that some components of Asian diets, such as fermented soy, spices, and vegetables, can impact the risk of CHD. The significance of the connections between diet and culture is exemplified by the Japanese emphasis on fruits, vegetables, and soy [49], and the Chinese focus on vegetable oil choices [50]. Considering the extensive dietary landscape in Asia, it is crucial to understand the complexities of different PDIs and to acknowledge the potential for synergistic or counteracting effects. This is essential for understanding the efficacy of plant-based diets in different Asian populations. To evaluate the robustness of our findings, we conducted various sensitivity analyses including adjustments for total animal food intake and energy intake from carbohydrates, proteins, and fats. In addition, our analyses excluded individuals who developed CHD within the initial 1-year and 2-year follow-up periods. Importantly, the association between a high hPDI and CHD did not change significantly, regardless of follow-up time and adjustment for these factors. The observed trend toward a reduction in CHD risk persisted, reinforcing the robustness of our findings.

This study had some limitations. First, the estimated risk proxy for CHD, which was calculated using the FRS, does not necessarily indicate the presence of CHD even though diagnostic data regarding CHD were included in this study. Second, since the FFQs did not represent absolute consumption, they may have introduced information bias; participants may have over-reported or under-reported their intake of certain foods. Third, while metabolic equivalents were used in the HEXA data, we only classified participants as active (physical activity for 30 minutes, at least once a day) or inactive. Fourth, the follow-up duration (median 4.2 years) might not be long enough to detect the development of a predicted risk of CHD. Finally, accurately determining the time of CHD onset was also hindered by the limited follow-up period.

Nonetheless, this study had notable strengths. To our knowledge, this is the first prospective investigation of the association between a plant-based diet and the predicted risk of CHD using the FRS and its components in Korean men. This study analyzed the dietary patterns of Korean men, providing valuable data that can inform future guidelines for plant-based dietary consumption in Korea. Furthermore, our analysis was strengthened by adjustment for potential key confounders, which increased the robustness of our findings about the independent association between plant-based diets and the risk of CHD. While these results provide valuable insights, additional prospective studies are needed to further elucidate the potential benefits of plant-based diets in the management of CHD.

In conclusion, this prospective cohort study highlights the role of high adherence to the hPDI in preventing CHD in Korean men. Further prospective studies are needed to determine the association between different types of plant-based diet indices and the risk of CHD in various Asian populations.

## Figures and Tables

**Figure f4-epih-46-e2024035:**
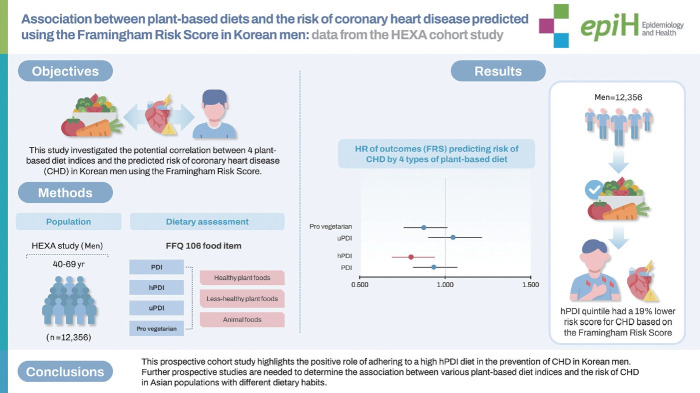


**Table 1.** The general characteristics of participants according to plant-based diet index quintiles^1^

Characteristics	PDI					p-value^2^
	Q1	Q2	Q3	Q4	Q5	
Men (n=12,356)	2,536	2,259	2,520	2,271	2,770	
Age (yr)	51.9±8.1	52.2±8.2	52.8±8.2	53.1±8.2	53.9±8.0	<0.001
BMI (kg/m^2^)	24.1±2.7	24.0±2.7	23.9±2.6	24.2±2.6	24.1±2.6	0.046
Weight						0.360
Underweight	38 (1.5)	36 (1.6)	40 (1.6)	26 (1.1)	39 (1.4)	
Normal	828 (32.7)	779 (34.5)	863 (34.3)	715 (31.5)	891 (32.2)	
Overweight	781 (30.8)	668 (29.6)	777 (30.8)	707 (31.1)	891 (32.2)	
Obese	889 (35.1)	776 (34.4)	840 (33.3)	823 (36.2)	949 (34.3)	
Income level (106 Korean won)						0.236
<3	1,171 (49.6)	982 (47.7)	1,104 (47.6)	974 (46.6)	1,166 (46.6)	
≥3	1,192 (50.4)	1,075(52.3)	1,215 (52.4)	1,116 (53.4)	1,338 (53.4)	
Education level						<0.001
Middle school or below	531 (21.2)	421 (18.8)	499 (20.0)	443 (19.7)	502 (18.3)	
High school	1,050 (41.9)	948 (42.4)	987 (39.6)	869 (38.6)	1,053 (38.4)	
College or above	928 (37.0)	866 (38.8)	1,007 (40.4)	937 (41.7)	1,187 (43.3)	
Alcohol consumption						<0.001
Non-drinker	561 (22.2)	579 (25.7)	643 (25.6)	630 (27.8)	876 (31.7)	
Current drinker	1,965 (77.8)	1,678 (74.4)	1,867 (74.4)	1,633 (72.2)	1,887 (68.3)	
Smoking status						<0.001
Never-smoker	718 (28.4)	698 (31.0)	837 (33.3)	769 (34.0)	983 (35.6)	
Past smoker	937 (37.1)	907 (40.2)	1,022 (40.6)	927 (41.0)	1,235 (44.7)	
Current smoker	873 (34.5)	650 (28.8)	656 (26.1)	564 (25.0)	544 (19.7)	
Physical activity						<0.001
Active	811 (32.8)	712 (32.4)	923 (37.6)	877 (39.9)	1,171 (43.9)	
Inactive	1,661 (67.2)	1,489 (67.7)	1,534 (62.4)	1,323 (60.2)	1,498 (56.1)	

Values are presented as mean±standard deviation or number (%). PDI, overall plant-based diet index; Q, quintile; BMI, body mass index.

1 Missing values are not shown in this table.

2 Using a generalized linear model for continuous variables and the chi-square test for categorical variables.

**Table 2.** Participants’ daily nutrient intake according to plant-based diet index quintiles (men: n=12,356)

Daily nutrient intake	Q1	Q2	Q3	Q4	Q5	p-value^1^
PDI						
Total energy intake (kcal/day)	1,711.7±434.2	1,783.8±440.8	1,860.6±473.9	1,954.0±511.0	2,035.1±518.3	<0.001
Energy from carbohydrates (%)	69.8±7.3	71.0±6.7	71.2±6.5	71.3±6.4	72.3±5.9	<0.001
Energy from protein (%)	13.3±2.6	13.2±2.5	13.3±2.4	13.4±2.4	13.4±2.3	<0.001
Energy from fat (%)	15.3±5.7	14.4±5.2	14.2±5.0	14.2±4.9	13.5±4.5	<0.001
Cholesterol (mg/day)	199.7±127.0	179.1±99.9	172.7±91.6	163.3±84.8	150.2±75.7	<0.001
Sodium (mg/day)	2,294.8±1,127.8	2,535.1±1,305.8	2,693.4±1,250.1	2,792.4±1,271.4	2,997.0±1,246.4	<0.001
Fiber (g/day)	4.6±1.6	5.3±2.0	5.7±1.9	6.1±2.1	6.8±2.2	<0.001
hPDI						
Total energy intake (kcal/day)	2,087.9±553.6	1,940.2±491.6	1,857.1±470.1	1,769.5±429.3	1,672.0±375.3	<0.001
Energy from carbohydrates (%)	67.2±6.4	69.8±6.2	71.3±6.2	73.1±5.7	74.9±5.7	<0.001
Energy from protein (%)	14.1±2.4	13.6±2.4	13.3±2.5	12.9±2.3	12.6±2.3	<0.001
Energy from fat (%)	17.7±4.9	15.5±4.7	14.1±4.7	12.7±4.4	11.1±4.3	<0.001
Cholesterol (mg/day)	202.2±103.7	182.3±90.5	173.5±98.4	157.6±90.4	142.1±97.8	<0.001
Sodium (mg/day)	2,783.7±1,186.0	2,726.8±1,207.0	2,691.7±1,300.5	2,611.1±1,311.8	2,506.6±1,293.2	<0.001
Fiber (g/day)	5.3±1.8	5.6±2.0	5.8±2.2	5.9±2.2	6.2±2.3	<0.001
uPDI						
Total energy intake (kcal/day)	2,152.8±522.8	1,967.7±469.9	1,863.7±455.8	1,729.7±403.5	1,603.8±392.2	<0.001
Energy from carbohydrates (%)	68.2±6.4	69.9±6.3	71.1±6.3	72.3±6.2	74.6±5.8	<0.001
Energy from protein (%)	14.6±2.5	13.9±2.3	13.3±2.3	12.8±2.2	11.9±2.0	<0.001
Energy from fat (%)	16.5±4.8	15.2±4.8	14.4±4.9	13.4±5.0	11.7±4.7	<0.001
Cholesterol (mg/day)	201.7±98.4	188.9±98.9	171.9±98.5	161.8±92.0	134.1±90.7	<0.001
Sodium (mg/day)	2,554.2±1,116.8	2,648.5±1,159.5	2,663.1±1,269.3	2,687.6±1,293.9	2,808.1±1,457.2	0.001
Fiber (g/day)	6.0±2.0	5.9±2.0	5.7±2.2	5.6±2.1	5.4±2.2	<0.001
Pro-vegetarian diet index						
Total energy intake (kcal/day)	1,788.9±469.6	1,833.7±479.5	1,872.5±499.0	1,933.4±495.6	1,937.1±497.4	<0.001
Energy from carbohydrates (%)	68.3±7.2	70.5±6.4	71.3±6.5	72.2±5.8	73.8±5.6	<0.001
Energy from protein (%)	13.8±2.7	13.4±2.5	13.3±2.4	13.2±2.3	12.9±2.2	<0.001
Energy from fat (%)	16.6±5.5	14.8±4.9	14.2±5.0	13.5±4.5	12.2±4.3	<0.001
Cholesterol (mg/day)	211.5±121.4	181.4±102.8	169.2±89.8	157.1±81.4	139.5±74.6	<0.001
Sodium (mg/day)	2,639.4±1,282.6	2,651.8±1,319.7	2,642.3±1,265.7	2,677.8±1,202.8	2,750.3±1,253.4	<0.001
Fiber (g/day)	5.0±1.9	5.4±2.0	5.7±2.1	6.1±2.1	6.6±2.2	<0.001

Values are presented as mean±standard deviation. PDI, overall plant-based diet index; hPDI, healthy plant-based diet index; uPDI, unhealthy plant-based diet index; Q, quintile.

1 Using a generalized linear model.

**Table 3.** Participants’ blood biomarker levels according to plant-based diet index quintiles (men: n=12,356)

Blood biomarker	Q1	Q2	Q3	Q4	Q5	p-value^1^
PDI						
TC (mg/dL)	194.6±32.4	193.4±30.8	192.1±32.3	192.0±30.8	192.3±32.1	<0.001
Triglyceride (mg/dL)	140.1±89.1	136.2±87.5	137.5±91.1	134.8±86.5	133.4±86.0	<0.001
FBG (mg/dL)	94.2±13.6	93.9±13.8	94.6±14.3	93.9±14.5	92.9±13.3	<0.001
HDL-C (mmol/L)	50.8±11.7	50.8±12.0	50.5±11.6	49.9±11.9	50.0±11.5	<0.001
LDL-C (mmol/L)	115.7±29.5	115.3±28.7	114.1±29.8	115.1±28.4	115.6±29.3	<0.001
Systolic blood pressure (mmHg)	122.9±13.0	123.0±13.2	122.9±13.1	122.3±13.4	123.1±13.3	<0.001
Diastolic blood pressure (mmHg)	77.4±9.2	77.5±9.5	77.4±9.0	77.0±9.2	77.5±9.2	<0.001
hPDI						
TC (mg/dL)	195.6±31.4	193.3±32.7	193.5±31.0	191.3±31.7	190.2±31.7	<0.001
Triglyceride (mg/dL)	140.7±88.2	139.3±92.4	137.0±88.2	133.4±85.3	130.7±85.8	<0.001
FBG (mg/dL)	93.5±12.8	93.8±13.8	93.7±13.6	94.0±14.1	94.5±15.4	<0.001
HDL-C (mmol/L)	50.1±11.7	50.5±11.9	50.5±11.7	50.3±11.5	50.6±11.9	<0.001
LDL-C (mmol/L)	117.3±29.0	114.9±29.6	115.5±29.3	114.3±28.9	113.4±28.7	<0.001
Systolic blood pressure (mmHg)	122.7±13.2	122.3±13.1	122.9±13.0	123.4±13.5	123.0±13.4	<0.001
Diastolic blood pressure (mmHg)	77.3±9.5	77.0±9.2	77.2±9.1	77.7±9.1	77.5±9.1	<0.001
uPDI						
TC (mg/dL)	194.8±31.3	194.9±32.0	193.2±31.6	191.7±31.6	189.4±31.9	<0.001
Triglyceride (mg/dL)	133.6±86.2	135.7±90.0	138.6±91.0	136.1±84.1	138.1±88.7	<0.001
FBG (mg/dL)	94.6±14.4	93.9±14.1	93.5±12.9	94.1±14.9	93.2±13.2	<0.001
HDL-C (mmol/L)	50.9±11.7	50.9±11.8	50.1±11.3	50.1±11.7	50.0±12.1	<0.001
LDL-C (mmol/L)	117.2±28.8	116.8±29.3	115.4±29.5	114.3±28.8	111.8±29.0	<0.001
Systolic blood pressure (mmHg)	123.1±13.2	122.9±12.9	122.9±13.1	122.7±13.5	122.7±13.4	<0.001
Diastolic blood pressure (mmHg)	77.7±9.0	77.2±9.2	77.4±9.2	77.2±9.5	77.2±9.2	<0.001
Pro-vegetarian diet index						
TC (mg/dL)	195.8±32.0	193.5±31.6	192.7±30.9	191.9±32.2	190.1±31.8	<0.001
Triglyceride (mg/dL)	139.3±90.6	142.0±94.7	132.7±78.6	136.5±90.7	133.0±88.4	<0.001
FBG (mg/dL)	94.2±14.5	93.8±12.8	94.2±13.4	93.9±14.9	93.1±13.6	<0.001
HDL-C (mmol/L)	50.4±11.5	50.8±12.2	50.8±11.7	50.1±11.6	49.9±11.8	<0.001
LDL-C (mmol/L)	117.5±29.6	114.3±29.5	115.3±28.3	114.5±29.4	113.6±29.1	<0.001
Systolic blood pressure (mmHg)	122.6±13.0	122.9±13.3	123.0±13.3	122.6±13.1	123.1±13.5	<0.001
Diastolic blood pressure (mmHg)	77.2±9.3	77.6±9.2	77.4±9.2	77.1±9.1	77.6±9.3	<0.001

Values are expressed as the mean±standard deviation. PDI, overall plant-based diet index; hPDI, healthy plant-based diet index; uPDI, unhealthy plant-based diet index; Q, quintile; TC, total cholesterol; FBG, fasting blood glucose; HDL-C, high-density lipoprotein cholesterol; LDL-C, low-density lipoprotein cholesterol.

1 Using a generalized linear model.

**Table 4.** Hazard ratios and 95% confidence intervals of coronary heart disease according to plant-based diet index quintiles (men: n=12,356)^1^

Variables	Q1	Q2	Q3	Q4	Q5	p for trend
PDI						
Person-years, mean (sum)	4.9 (12,309.0)	5.0 (11,370.3)	5.0 (12,528.8)	5.0 (11,399.3)	5.0 (13,948.4)	
Score median (range)	44 (32-46)	48 (47-49)	51 (50-52)	54 (53-56)	58 (56-71)	
Case (n)	401	349	405	384	478	
Model 1	1.00 (reference)	0.93 (0.80, 1.07)	0.88 (0.76, 1.01)	0.86 (0.74, 0.98)	0.86 (0.75, 0.98)	0.017
Model 2	1.00 (reference)	0.96 (0.83, 1.11)	0.91 (0.79, 1.04)	0.89 (0.77, 1.03)	0.93 (0.81, 1.07)	0.889
Model 3	1.00 (reference)	0.94 (0.82, 1.09)	0.91 (0.79, 1.04)	0.91 (0.78, 1.04)	0.93 (0.81, 1.07)	0.859
hPDI						
Person-years, mean (sum)	4.8 (12,964.2)	4.9 (11,670.7)	5.0 (13,371.9)	5.0 (11,850.2)	5.1 (11,561.8)	
Score median (range)	42 (27-45)	48 (46-49)	51 (50-53)	55 (54-57)	60 (58-77)	
Case (n)	345	333	460	455	424	
Model 1	1.00 (reference)	0.84 (0.72, 0.98)	0.67 (0.75, 1.00)	0.89 (0.77, 1.02)	0.75 (0.65, 0.86)	0.001
Model 2	1.00 (reference)	0.87 (0.75, 1.01)	0.92 (0.80, 1.07)	0.90 (0.78, 1.04)	0.81 (0.69, 0.94)	0.013
Model 3	1.00 (reference)	0.88 (0.75, 1.02)	0.95 (0.82, 1.10)	0.92 (0.80, 1.07)	0.80 (0.69, 0.93)	0.010
uPDI						
Person-years, mean (sum)	4.9 (13,124.0)	4.9 (11,951.4)	5.0 (13,047.6)	5.0 (11,290.8)	5.1 (12,005.0)	
Score median (range)	43 (28-45)	48 (46-49)	52 (50-53)	55 (54-57)	61 (58-75)	
Case (n)	416	389	416	353	443	
Model 1	1.00 (reference)	1.02 (0.89, 1.18)	1.03 (0.90, 1.18)	1.00 (0.86, 1.15)	1.09 (0.95, 1.25)	0.266
Model 2	1.00 (reference)	1.00 (0.87, 1.15)	1.05 (0.91, 1.20)	1.00 (0.87, 1.17)	1.08 (0.93, 1.25)	0.315
Model 3	1.00 (reference)	0.99 (0.86, 1.14)	1.02 (0.89, 1.18)	0.99 (0.85, 1.15)	1.05 (0.90, 1.21)	0.549
Pro-vegetarian diet index						
Person-years, mean (sum)	5.0 (13,258.2)	4.9 (9,223.4)	5.0 (15,500.2)	5.0 (12,927.9)	5.0 (10,509.1)	
Score median (range)	27 (15-29)	31 (30-31)	33 (32-34)	36 (35-37)	39 (38-50)	
Case (n)	400	303	511	436	367	
Model 1	1.00 (reference)	1.07 (0.92, 1.25)	0.92 (0.80, 1.04)	0.93 (0.81, 1.06)	0.84 (0.73, 0.97)	0.007
Model 2	1.00 (reference)	1.08 (0.93, 1.25)	0.93 (0.82, 1.06)	0.93 (0.81, 1.07)	0.87 (0.75, 1.01)	0.384
Model 3	1.00 (reference)	1.06 (0.91, 1.23)	0.94 (0.82, 1.07)	0.94 (0.82, 1.08)	0.88 (0.76, 1.01)	0.427

Values are presented as hazard ratio (95% confidence interval). PDI, overall plant-based diet index; hPDI, health plant-based diet index; uPDI, unhealthy plant-based diet index; Q, quintile.

1 Model 1: Adjusted for age and body mass index (kg/m^2^); Model 2: Additionally adjusted for education level (middle school or below, high school, or college or above), smoking status (never, past, or current smoker), alcohol consumption (non-drinker or current drinker), household income level (<3 or ≥3 million Korean won/mo), physical activity (yes or no), and energy intake; Model 3: Additionally adjusted for age, waist circumference, education level (middle school or below, high school, or college or above), smoking status (never, past, or current smoker), alcohol consumption (non-drinker or current drinker), household income level (<3 or ≥3 million Korean won/mo), physical activity (yes or no), and energy intake.

## Supplementary materials

Supplementary Material 1. Food items constituting food groups based on the Korean Genome and Epidemiology Study (KoGES)

Supplementary Material 2. General characteristics of participants according to healthy plant-based diet index quintile

Supplementary Material 3. General characteristics of participants according to unhealthy plant-based diet index quintile

Supplementary Material 4. General characteristics of participants according to unhealthy pro-vegetarian diet index quintile

Supplementary Material 5. Hazard ratios (HRs) and 95% confidence intervals (CI) for CHD based on plant-based diet index quintiles after controlling for additional variables.
