# Supplementary material for: Association between plant-based diets and the risk of coronary heart disease predicted using the Framingham Risk Score in Korean men: data from the HEXA cohort study
Source: Epidemiol Health. 2024 Feb 28;46:e2024035. doi: 10.4178/epih.e2024035 (PMC11176718; doi:10.4178/epih.e2024035)
Supplement: Supplementary Material 1. — Food items constituting food groups based on the Korean Genome and Epidemiology Study (KoGES) [file epih-46-e2024035-Supplementary-1.docx]

**Supplementary Material 1.** Food items constituting food groups based on the Korean Genome and Epidemiology Study (KoGES)

| **Food groups** | **Items in the food frequency questionnaire** | **PDI** | **hPDI** | **uPDI** | **Pro-vegetarian** |
| --- | --- | --- | --- | --- | --- |
| **Healthy plant foods** | | | | |  |
| Whole grains | Cooked rice with soybean, cooked rice with other cereals | + | + | - | +^1^ |
| Fruits | Persimmon/dried persimmon, tangerine, oriental melon/melon, banana, pear/pear juice, apple/apple juice, orange/orange juice, watermelon, peach/plum, strawberry, grape/grape juice | + | + | - | + |
| Vegetables | Green pepper, green pepper leaf/chamnamul, spinach, lettuce, perilla leaves, crown daisy/chives/watercress, other green vegetable, radish, sesame leaves/vegetable salad, Deodeck/ bellflower root, onion, napa cabbage/napa cabbage soup, cucumber, bean sprouts/mung-bean sprouts, carrot/carrot juice, pumpkin/kabocha squash, zucchini, bracken/sweet potato stem, oyster mushrooms, other mushrooms, tomato/tomato juice/tomato sauce, laver, kelp/seaweed | + | + | - | + |
| Nuts | Peanuts/almonds/pine nuts | + | + | - | + |
| Legumes | Beans/beans cooked in soy sauce, tofu, soybean milk | + | + | - | + |
| Tea & Coffee | Green tea, coffee | + | + | - | Not scored |
| **Less-healthy plant foods** | | | | |  |
| Refined grains | Cooked rice (well-milled), roasted grain powder, instant noodles, hot noodles, black bean sauce noodles, cold noodles, cold noodles, rice cake/rice cake soup, other rice cakes, white breads, other breads, cereals, stir-fried noodles and vegetables, starch jelly | + | - | + | +^1^ |
| Potatoes | Potatoes, sweet potatoes | + | - | + | + |
| Sugar-sweetened beverages | Soft drinks (coke/cider), other beverages (sweetened rice tea, citron tea) | + | - | + | Not scored |
| Sweets and Desserts | Sweet red bean bread, cake/chocolate pie, snacks, candies/chocolate, sugars (added to tea or coffee) | + | - | + | Not scored |
| Salty plant foods | Bean paste/bean paste soup, Kimchi (Korean cabbage, radish), radish kimchi (preserved in soy sauce or salt), watery radish kimchi, other kimchi, pickled vegetables (preserved in soy sauce or salt) | + | - | + | Not scored |
| **Animal foods** | | | | |  |
| Animal fat | Butter, cream (added to tea or coffee) | - | - | - | - |
| Dairy | Milk, ice cream, yogurt/Yoplait, cheese | - | - | - | - |
| Eggs | Eggs/quail eggs | - | - | - | - |
| Fish or Seafood | Sashimi, belt fish, eel, yellow croaker/sea bream/sole, Alaska pollack/frozen pollack/dried pollack, mackerel/pacific saury, anchovy/stir-fried anchovy, squid/dried squid/octopus, canned tuna, fishcakes, crab/marinated crab, clam/sea snail, oyster,  shrimp, salted shrimp/salted fish | - | - | - | - |
| Meat | Dog meat, chicken (fried, stew, braised spicy chicken), grilled pork/stir-fried pork/pork bulgogi/Korean meatball, pork belly, steamed pork, processed meat (ham, sausage), steak/grilled beef, beef soup, beef stew, organ meat | - | - | - | - |
| Miscellaneous animal foods | Dumplings, pizza/hamburger | - | - | - | Not scored |

PDI, overall plant-based diet index; hPDI, healthy plant-based diet index; uPDI, unhealthy plant-based diet index

The PDI, hPDI, uPDI, and pro-vegetarian diet index were categorized into three groups: “Healthy plant foods,” “Less-healthy plant foods,” and “Animal foods.”

“+” Positive scores indicate that participants in the highest food-group quintile received a score of 5.

“–” Negative scores indicate that participants in the highest food-group quintile received a score of 1.

^1^Whole and refined grains were rated by combining them into a “grains” food group in the pro-vegetarian diet index.
