# Supplementary material for: Association between plant-based diets and the risk of coronary heart disease predicted using the Framingham Risk Score in Korean men: data from the HEXA cohort study
Source: Epidemiol Health. 2024 Feb 28;46:e2024035. doi: 10.4178/epih.e2024035 (PMC11176718; doi:10.4178/epih.e2024035)
Supplement: Supplementary Material 2. — General characteristics of participants according to healthy plant-based diet index quintile [file epih-46-e2024035-Supplementary-2.docx]

**Supplementary Material 2*.*** General characteristics of participants according to healthy plant-based diet index quintile

|  | **hPDI** | | | | | **P-value^1^** |
| --- | --- | --- | --- | --- | --- | --- |
|  | **Q1** | **Q2** | **Q3** | **Q4** | **Q5** |  |
| **Men (N = 12,356)** | 2679 | 2360 | 2694 | 2348 | 2275 |  |
| **Age (years)** | 49.3 (7.8) | 51.5 (7.9) | 53.4 (7.9) | 54.4 (7.9) | 56.0 (7.5) | <0.0001 |
| **BMI (kg/m^2^)** | 24.2 (2.8) | 24.1 (2.7) | 24.1 (2.6) | 24.1 (2.6) | 23.8 (2.5) | <0.0001 |
| **Obesity, n (%)** |  | | | | | |
| Underweight | 37 (1.4) | 31 (1.3) | 36 (1.3) | 26 (1.1) | 49 (2.2) | 0.0004 |
| Normal | 862 (32.2) | 758 (32.1) | 861 (32.0) | 798 (34.0) | 797 (35.0) |  |
| Overweight | 788 (29.4) | 744 (31.5) | 842 (31.3) | 716 (30.5) | 734 (32.3) |  |
| Obese | 992 (37.0) | 827 (35.0) | 955 (35.5) | 808 (34.4) | 695 (30.6) |  |
| **Income level, n (%)** |  | | | | | |
| < 3 million won | 1131 (44.9) | 1039 (47.9) | 1168 (47.8) | 1040 (48.9) | 1019 (49.2) | 0.0233 |
| ≥ 3 million won | 1391 (55.2) | 1129 (52.1) | 1276 (52.2) | 1088 (51.1) | 1052 (50.8) |  |
| **Education level, n (%)** |  | | | | | |
| Middle school or below | 418 (15.7) | 477 (20.5) | 543 (20.4) | 499 (21.4) | 459 (20.4) | <0.0001 |
| High school | 1123 (42.2) | 935 (40.1) | 1079 (40.6) | 923 (39.7) | 847 (37.7) |  |
| College or above | 1122 (42.1) | 921 (39.5) | 1035 (39.0) | 905 (38.9) | 942 (41.9) |  |
| **Alcohol consumption, n (%)** |  | | | | | |
| Non-drinker | 603 (22.6) | 618 (26.2) | 711 (26.5) | 648 (27.7) | 709 (31.3) | <0.0001 |
| Current drinker | 2066 (77.4) | 1737 (73.8) | 1971(73.5) | 1696 (72.4) | 1560 (68.8) |  |
| **Smoking status, n (%)** |  | | | | | |
| Never-smoker | 714 (26.7) | 695 (29.5) | 911 (33.9) | 806 (34.4) | 879 (38.7) | <0.0001 |
| Past smoker | 913 (34.2) | 932 (39.6) | 1102 (41.1) | 1024 (43.7) | 1057 (46.6) |  |
| Current smoker | 1044 (39.1) | 726 (30.9) | 671 (25.0) | 512 (21.9) | 334 (14.7) |  |
| **Physical activity, n (%)** |  | | | | | |
| Active | 790 (30.2) | 776 (33.9) | 1008 (38.6) | 930 (40.8) | 990 (45.0) | <0.0001 |
| Inactive | 1830 (69.9) | 1512 (66.1) | 1605 (61.4) | 1350 (59.2) | 1208 (55.0) |  |

hPDI, healthy plant-based diet index; Q, quintile; BMI, body mass index

^1^Values are expressed as the mean (SD) or *n* (%); *P*-values< 0.05 were calculated using a generalized linear model for continuous variables and the chi-square test for categorical variables.

Missing values are not shown in this table.
