# Supplementary material for: Association between plant-based diets and the risk of coronary heart disease predicted using the Framingham Risk Score in Korean men: data from the HEXA cohort study
Source: Epidemiol Health. 2024 Feb 28;46:e2024035. doi: 10.4178/epih.e2024035 (PMC11176718; doi:10.4178/epih.e2024035)
Supplement: Supplementary Material 3. — General characteristics of participants according to unhealthy plant-based diet index quintile [file epih-46-e2024035-Supplementary-3.docx]

**Supplementary Material 3*.*** General characteristics of participants according to unhealthy plant-based diet index quintile

|  | **uPDI** | | | | | **P-value^1^** |
| --- | --- | --- | --- | --- | --- | --- |
|  | **Q1** | **Q2** | **Q3** | **Q4** | **Q5** |  |
| **Men (N = 12,356)** | 2660 | 2445 | 2619 | 2276 | 2356 |  |
| **Age (years)** | 52.8 (7.8) | 52.8 (8.2) | 52.4 (8.1) | 52.7 (8.2) | 53.4 (8.3) | 0.0012 |
| **BMI (kg/m^2^)** | 24.3 (2.6) | 24.1 (2.6) | 24.1 (2.6) | 24.0 (2.7) | 23.8 (2.7) | <0.0001 |
| **Obesity, n (%)** |  | | | | | |
| Underweight | 20 (0.8) | 28 (1.2) | 40 (1.5) | 39 (1.7) | 52 (2.2) | <0.0001 |
| Normal | 800 (30.1) | 766 (31.3) | 850 (32.5) | 794 (34.9) | 866 (36.8) |  |
| Overweight | 852 (32.0) | 776 (31.7) | 828 (31.6) | 673 (29.6) | 695 (29.5) |  |
| Obese | 988 (37.1) | 875 (35.8) | 901 (34.4) | 770 (33.8) | 743 (31.5) |  |
| **Income level, n (%)** |  | | | | | |
| < 3 million won | 986 (39.4) | 988 (43.4) | 1121 (47.1) | 1039 (49.9) | 1263 (60.4) | <0.0001 |
| ≥ 3 million won | 1515 (60.6) | 1291 (56.7) | 1259 (52.9) | 1044 (50.1) | 827 (39.6) |  |
| **Education level, n (%)** |  | | | | | |
| Middle school or below | 298 (11.3) | 378 (15.6) | 500 (19.3) | 494 (22.0) | 726 (31.2) | <0.0001 |
| High school | 1001 (37.9) | 947 (39.1) | 1067 (41.2) | 931 (41.5) | 961 (41.3) |  |
| College or above | 1343 (50.8) | 1097 (45.3) | 1025 (39.5) | 821 (36.6) | 639 (27.5) |  |
| **Alcohol consumption, n (%)** |  | | | | | |
| Non-drinker | 604 (22.8) | 639 (26.2) | 664 (25.4) | 647 (28.5) | 735 (31.3) | <0.0001 |
| Current drinker | 2048 (77.2) | 1801 (73.8) | 1946 (74.6) | 1622 (71.5) | 1613 (68.7) |  |
| **Smoking status, n (%)** |  | | | | | |
| Never-smoker | 869 (32.8) | 805 (33.0) | 841 (32.0) | 749 (33.1) | 741 (31.5) | <0.0001 |
| Past smoker | 1166 (44.0) | 1027 (42.1) | 1058 (40.5) | 887 (39.1) | 890 (37.8) |  |
| Current smoker | 614 (23.2) | 608 (24.9) | 714 (27.3) | 630 (27.8) | 721 (30.7) |  |
| **Physical activity, n (%)** |  | | | | | |
| Active | 1252 (48.7) | 990 (41.8) | 907 (35.7) | 737 (33.2) | 608 (26.4) | <0.0001 |
| Inactive | 1318 (51.3) | 1381 (58.3) | 1634 (64.3) | 1480 (66.8) | 1692 (73.6) |  |

uPDI, unhealthy plant-based diet index; Q, quintile; BMI, body mass index

^1^Values are expressed as the mean (SD) or *n* (%); *P*-values< 0.05 were calculated using a generalized linear model for continuous variables and the chi-square test for categorical variables.

Missing values are not shown in this table.
