# Supplementary material for: Association between plant-based diets and the risk of coronary heart disease predicted using the Framingham Risk Score in Korean men: data from the HEXA cohort study
Source: Epidemiol Health. 2024 Feb 28;46:e2024035. doi: 10.4178/epih.e2024035 (PMC11176718; doi:10.4178/epih.e2024035)
Supplement: Supplementary Material 4. — General characteristics of participants according to unhealthy pro-vegetarian diet index quintile [file epih-46-e2024035-Supplementary-4.docx]

**Supplementary Material 4*.*** General characteristics of participants according to unhealthy pro-vegetarian diet index quintile

|  | **Pro-vegetarian diet index** | | | | | **P-value^1^** |
| --- | --- | --- | --- | --- | --- | --- |
|  | **Q1** | **Q2** | **Q3** | **Q4** | **Q5** |  |
| **Men (N = 12,356)** | 2677 | 1877 | 3101 | 2604 | 2097 |  |
| **Age (years)** | 50.8 (8.0) | 52.1 (8.1) | 53.0 (8.1) | 53.6 (8.0) | 54.9 (7.9) | 0.2435 |
| **BMI (kg/m^2^)** | 24.1 (2.7) | 24.0 (2.7) | 24.0 (2.6) | 24.1 (2.6) | 24.0 (2.6) |  |
| **Obesity, n (%)** |  | | | | | |
| Underweight | 44 (1.6) | 26 (1.4) | 35 (1.1) | 33(1.3) | 41 (2.0) | 0.1292 |
| Normal | 854 (31.9) | 633 (33.7) | 1063 (34.3) | 838 (32.2) | 688 (32.8) |  |
| Overweight | 830 (31.0) | 545 (29.0) | 954 (30.8) | 821 (31.5) | 674 (32.1) |  |
| Obese | 949 (35.5) | 673 (35.9) | 1049 (33.8) | 912 (35.0) | 694 (33.1) |  |
| **Income level, n (%)** |  | | | | | |
| < 3 million won | 1141 (46.0) | 841 (48.5) | 1310 (46.4) | 1126 (47.1) | 979 (51.3) | 0.0035 |
| ≥ 3 million won | 1338 (54.0) | 892 (51.5) | 1515 (53.6) | 1263 (52.9) | 928 (48.7) |  |
| **Education level, n (%)** |  | | | | | |
| Middle school or below | 474 (17.9) | 346 (18.7) | 669 (21.8) | 497 (19.3) | 410 (19.8) | <0.0001 |
| High school | 1140 (43.1) | 792 (42.7) | 1182 (38.5) | 1026 (39.8) | 767 (37.0) |  |
| College or above | 1033 (39.0) | 717 (38.7) | 1223 (39.8) | 1054 (40.9) | 898 (43.3) |  |
| **Alcohol consumption, n (%)** |  | | | | | |
| Non-drinker | 604 (22.6) | 444 (23.7) | 794 (25.8) | 734 (28.2) | 713 (34.1) | <0.0001 |
| Current drinker | 2067 (77.4) | 1427 (76.3) | 2290 (74.3) | 1866 (71.8) | 1380 (65.9) |  |
| **Smoking status, n (%)** |  | | | | | |
| Never-smoker | 699 (26.2) | 541 (28.9) | 1023 (33.1) | 919 (35.4) | 823 (39.4) | <0.0001 |
| Past smoker | 943 (35.3) | 732 (39.0) | 1308 (42.3) | 1109 (42.7) | 936 (44.8) |  |
| Current smoker | 1027 (38.5) | 602 (32.1) | 758 (24.5) | 568 (21.9) | 332 (15.9) |  |
| **Physical activity, n (%)** |  | | | | | |
| Active | 811 (31.0) | 640 (35.1) | 1118 (37.1) | 1015 (40.3) | 910 (44.9) | <0.0001 |
| Inactive | 1802 (69.0) | 1186 (65.0) | 1899 (62.9) | 1502 (59.7) | 1116 (55.1) |  |

Q, quintile; BMI, body mass index

^1^Values are expressed as the mean (SD) or *n* (%); *P*-values< 0.05 were calculated using a generalized linear model for continuous variables and the chi-square test for categorical variables.

Missing values are not shown in this table.
