# Supplementary material for: Association between plant-based diets and the risk of coronary heart disease predicted using the Framingham Risk Score in Korean men: data from the HEXA cohort study
Source: Epidemiol Health. 2024 Feb 28;46:e2024035. doi: 10.4178/epih.e2024035 (PMC11176718; doi:10.4178/epih.e2024035)
Supplement: Supplementary Material 5. — Hazard ratios (HRs) and 95% confidence intervals (CI) for CHD based on plant-based diet index quintiles after controlling for additional variables. [file epih-46-e2024035-Supplementary-5.docx]

**Supplementary Material 5.** Hazard ratios (HRs) and 95% confidence intervals (CI) for CHD based on plant-based diet index quintiles after controlling for additional variables.

| **PDI** | Q1 | Q2 | Q3 | Q4 | Q5 | *P* for trend |
| --- | --- | --- | --- | --- | --- | --- |
| **Men (N=12,356)** |  |  |  |  |  |  |
| Person-years, mean (sum) | 4.9 (12309.0) | 5.0 (11370.3) | 5.0 (12528.8) | 5.0 (11399.3) | 5.0 (13948.4) |  |
| Score median (range) | 44 (32–46) | 48 (47–49) | 51 (50–52) | 54 (53–56) | 58 (56–71) |  |
| Case, n | 401 | 349 | 405 | 384 | 478 |  |
| Model 1 | 1.000 (ref.) | 0.926 (0.802–1.069) | 0.879 (0.765–1.009) | 0.856 (0.744–0.985) | 0.859 (0.752–0.981) | 0.0167 |
| Model 2 | 1.000 (ref.) | 0.958 (0.829–1.107) | 0.908 (0.790–1.043) | 0.890 (0.772–1.027) | 0.930 (0.811–1.067) | 0.8890 |
| Model 3 | 1.000 (ref.) | 0.945 (0.818–1.092) | 0.906 (0.788–1.042) | 0.906 (0.785–1.044) | 0.932 (0.812–1.069) | 0.8592 |
| Model 4 | 1.000 (ref.) | 0.954 (0.825–1.102) | 0.902 (0.784–1.037) | 0.884 (0.766–1.02) | 0.920 (0.801–1.057) | 0.8819 |
| Model 5 | 1.000 (ref.) | 0.96 (0.831–1.11) | 0.916 (0.796–1.055) | 0.905 (0.783–1.045) | 0.952 (0.826–1.097) | 0.9945 |
| **hPDI** |  |  |  |  |  |  |
| Person-years, mean (sum) | 4.8 (12964.2) | 4.9 (11670.7) | 5.0 (13371.9) | 5.0 (11850.2) | 5.1 (11561.8) |  |
| Score median (range) | 42 (27–45) | 48 (46–49) | 51 (50–53) | 55 (54–57) | 60 (58–77) |  |
| Case, n | 345 | 333 | 460 | 455 | 424 |  |
| Model 1 | 1.000 (ref.) | 0.842 (0.724–0.979) | 0.867 (0.753–0.998) | 0.889 (0.772–1.024) | 0.747 (0.647–0.864) | 0.0006 |
| Model 2 | 1.000 (ref.) | 0.869 (0.747–1.011) | 0.924 (0.802–1.066) | 0.901 (0.780–1.041) | 0.807 (0.695–0.938) | 0.0134 |
| Model 3 | 1.000 (ref.) | 0.878 (0.755–1.022) | 0.951 (0.825–1.096) | 0.923 (0.799–1.066) | 0.801 (0.689–0.930) | 0.0105 |
| Model 4 | 1.000 (ref.) | 0.865 (0.743–1.007) | 0.918 (0.795–1.06) | 0.894 (0.772–1.034) | 0.797 (0.684–0.930) | 0.0104 |
| Model 5 | 1.000 (ref.) | 0.869 (0.746–1.012) | 0.923 (0.798–1.067) | 0.894 (0.77–1.037) | 0.798 (0.681–0.937) | 0.0132 |
| **uPDI** |  |  |  |  |  |  |
| Person-years, mean (sum) | 4.9 (13124.0) | 4.9 (11951.4) | 5.0 (13047.6) | 5.0 (11290.8) | 5.1 (12005.0) |  |
| Score median (range) | 43 (28-45) | 48 (46–49) | 52 (50–53) | 55 (54–57) | 61 (58–75) |  |
| Case, n | 416 | 389 | 416 | 353 | 443 |  |
| Model 1 | 1.000 (ref.) | 1.025 (0.893–1.178) | 1.029 (0.898–1.179) | 0.997 (0.865–1.150) | 1.092 (0.955–1.249) | 0.2656 |
| Model 2 | 1.000 (ref.) | 0.999 (0.869–1.150) | 1.047 (0.910–1.205) | 1.005 (0.866–1.166) | 1.079 (0.931–1.251) | 0.3147 |
| Model 3 | 1.000 (ref.) | 0.989 (0.860–1.138) | 1.025 (0.890–1.179) | 0.992 (0.855–1.151) | 1.046 (0.901–1.213) | 0.5489 |
| Model 4 | 1.000 (ref.) | 1.007 (0.872–1.163) | 1.058 (0.912–1.228) | 1.02 (0.867–1.199) | 1.099 (0.928–1.300) | 0.2639 |
| Model 5 | 1.000 (ref.) | 1.013 (0.879–1.167) | 1.069 (0.926–1.233) | 1.035 (0.887–1.207) | 1.120 (0.956–1.313) | 0.1545 |
| **Pro-vegetarian diet index** | |  |  |  |  |  |
| Person-years, mean (sum) | 5.0 (13258.2) | 4.9 (9223.4) | 5.0 (15500.2) | 5.0 (12927.9) | 5.0 (10509.1) |  |
| Score median (range) | 27 (1529) | 31 (30–31) | 33 (32–34) | 36 (35–37) | 39 (38–50) |  |
| Case, n | 400 | 303 | 511 | 436 | 367 |  |
| Model 1 | 1.000 (ref.) | 1.073 (0.924–1.246) | 0.917 (0.804–1.045) | 0.926 (0.808–1.060) | 0.844 (0.731–0.973) | 0.0074 |
| Model 2 | 1.000 (ref.) | 1.076 (0.926–1.251) | 0.953 (0.834–1.088) | 1 (0.871–1.147) | 0.944 (0.816–1.093) | 0.3845 |
| Model 3 | 1.000 (ref.) | 1.055 (0.908–1.226) | 0.96 (0.841–1.097) | 1.005 (0.875–1.153) | 0.943 (0.815–1.091) | 0.4268 |
| Model 4 | 1.000 (ref.) | 1.074 (0.923–1.25) | 0.951 (0.832–1.088) | 0.997 (0.867–1.147) | 0.941 (0.810–1.093) | 0.3624 |
| Model 5 | 1.000 (ref.) | 1.078 (0.926–1.256) | 0.957 (0.835–1.097) | 1.005 (0.871–1.161) | 0.954 (0.816–1.115) | 0.4829 |

PDI: overall plant-based diet index, hPDI: health plant-based diet index, uPDI: unhealthy plant-based diet index, Q: quintile.

Model 1 was adjusted for age and body mass index (kg/m^2^). Model 2 was additionally adjusted for age, body mass index (kg/m^2^), education level (middle school or below, high school, or college or above), smoking status (never, past, or current smoker), alcohol consumption (non-drinker or current drinker), household income level (<3 million won or ≥3 million won per month), physical activity (yes or no), and energy intake.

Model 3 was additionally adjusted for age, waist circumference, education level (middle school or below, high school, or college or above), smoking status (never, past, or current smoker), alcohol consumption (non-drinker or current drinker), household income level (<3 million won or ≥3 million won per month), physical activity (yes or no), and energy intake.

Model 4 was additionally adjusted for age, body mass index (kg/m^2^), education level (middle school or below, high school, or college or above), smoking status (never, past, or current smoker), alcohol consumption (non-drinker or current drinker), household income level (<3 million won or ≥3 million won per month), physical activity (yes or no), energy intake, and animal food intake.

Model 5 was additionally adjusted for age, body mass index (kg/m^2^), education level (middle school or below, high school, or college or above), smoking status (never, past, or current smoker), alcohol consumption (non-drinker or current drinker), household income level (<3 million won or ≥3 million won per month), physical activity (yes or no), and energy intake from carbohydrates, proteins, or fat.
